# Genotype-first approach reveals monogenic lipodystrophy is underdiagnosed, with health and mortality risks

**DOI:** 10.1016/j.ebiom.2026.106255

**Published:** 2026-04-18

**Authors:** Luke N. Sharp, Kevin Colclough, Jacques Murray Leech, Amy V. Evans, Andrew T. Hattersley, Michael N. Weedon, Rebecca J. Brown, Kashyap A. Patel

**Affiliations:** a Department of Clinical and Biomedical Sciences, Faculty of Health and Life Sciences, University of Exeter, Exeter, UK; b Exeter Genomic Laboratory, Royal Devon University Healthcare NHS Foundation Trust, Exeter, UK; c Centre for Endocrinology, Barts and The London School of Medicine, Queen Mary University of London, London, UK; d National Institute of Diabetes and Digestive and Kidney Diseases, National Institute of Health, Bethesda, USA

**Keywords:** Lipodystrophy, Monogenic diabetes, Genetics

## Abstract

**Background:**

Monogenic lipodystrophy is a metabolic disorder that predisposes to diabetes and cardiovascular disease, yet its true prevalence and clinical spectrum remain uncertain. We used a genotype-first approach with an aim to estimate the prevalence, phenotypic spectrum, risk of cardiometabolic disorders and all-cause mortality associated with monogenic lipodystrophy in the population. We also assessed how these clinically unselected cases differ from clinically identified cases.

**Methods:**

We analysed whole-genome sequencing data from 490,414 UK Biobank participants to identify pathogenic variants in 23 lipodystrophy genes. Individuals carrying a pathogenic genotype were compared with non-carriers for anthropometric traits, metabolic biomarkers, cardiometabolic outcomes, and all-cause mortality. We also compared UK Biobank cases with a clinically identified cohort of 58 individuals with monogenic lipodystrophy.

**Findings:**

We identified 31 carriers of pathogenic monogenic lipodystrophy variants, giving a prevalence of 1 in 15,820 (95% CI: 1 in 23,282–1 in 11,145). Variants in *PPARG* and *LMNA* were most frequent, and prevalence did not differ by sex (*P* = 0.37). Compared with non-carriers, carriers had similar BMI but lower total body fat percentage (24.7% vs. 31.4%, *P* = 1.25 × 10^−5^), higher waist–hip ratio adjusted for BMI (0.91 vs. 0.87, *P* = 0.0044), elevated triglycerides (2.9 vs. 1.7 mmol/L, *P* = 6.69 × 10^−5^), and reduced HDL cholesterol (0.99 vs. 1.45 mmol/L, *P* = 6.26 × 10^−9^). These features were similar between men and women. None of the carriers had a diagnosis of lipodystrophy in electronic health records. Carriers had increased risk of diabetes (Adjusted HR 4.41, 95% CI 2.5–7.76), coronary artery disease (Adjusted HR 2.97, 95% CI 1.42–6.24), and heart failure (Adjusted HR 5.28, 95% CI 2.52–11.07). They also had almost fourfold higher mortality (Adjusted HR 4.02, 95% CI 2.16–7.48) over a mean follow-up of 13.6 years. Compared with clinically identified cases, UK Biobank carriers had milder phenotypes.

**Interpretation:**

Monogenic lipodystrophy is more common than currently recognised and most cases remain undiagnosed despite significant cardiometabolic and mortality risks. These findings highlight the value of genotype-first approaches in studying lipodystrophy and support the need for earlier recognition and treatment in clinical practice.

**Funding:**

This work is funded by 10.13039/501100000361Diabetes UK (19/0005994 and 21/0006335), the MRC (MR/T00200X/1) and the 10.13039/100010269Wellcome Trust (219606/Z/19/Z).

**Research in context**

**Evidence before this study**

Monogenic lipodystrophy is regarded as extremely rare, with prevalence estimates of approximately 1–5 cases per million derived mainly from electronic health records and clinically recognised cases. However, lipodystrophy phenotype vary widely and overlap with common cardiometabolic conditions, particularly in partial lipodystrophy. As a result, they likely underestimate true prevalence and provide limited insight into penetrance, cardiometabolic risk, and mortality. One previous genotype-first study suggested a higher prevalence, but its modest sample size and strong founder effect limited generalisability.

**Added value of this study**

Using whole-genome sequencing data from nearly 500,000 UK Biobank participants, We show that monogenic lipodystrophy affects approximately one in 15,000 individuals in the general population, around ten-fold higher than previous phenotype-based estimates, with no difference in prevalence between males and females. Although these individuals have milder and overlapping clinical features compared with clinically ascertained cases, they show substantial excess risk, with two- to five-fold increases in lipodystrophy-related cardiometabolic outcomes and an almost four-fold increase in all-cause mortality. None had a recorded diagnosis of lipodystrophy, indicating that current clinical practice fails to identify most affected individuals.

**Implications of all the available evidence**

These findings show that monogenic lipodystrophy is markedly underdiagnosed, spans a broader and milder phenotypic spectrum than currently recognised, and carries a substantial burden of cardiometabolic disease and premature mortality even in clinically unselected individuals. Together with existing evidence, our results support wider awareness and the development of tools that enable earlier identification, with the potential to improve survival in this overlooked population.

## Introduction

A diagnosis of lipodystrophy has significant clinical implications. Lipodystrophy is a heterogeneous metabolic disorder characterised by abnormal fat distribution.^1^, ^2^, ^3^ In affected individuals, fat accumulates preferentially in visceral rather than subcutaneous tissues, leading to serious metabolic complications such as diabetes and cardiovascular disease.^1^, ^2^, ^3^, ^4^ Lipodystrophy can be either acquired, polygenic or monogenic, with pathogenic variants in over 20 genes known to cause the latter.^1^, ^2^, ^3^ Monogenic lipodystrophy presents as either generalised or partial, depending on the amount and distribution of fat loss and underlying genetic cause.^1^, ^2^, ^3^ Early diagnosis and intervention are important to reduce long-term metabolic complications.

Despite its clinical relevance, the prevalence and phenotypic spectrum of monogenic lipodystrophy in the general population remains poorly characterised. Accurate estimates are critical for informing genetic testing strategies, guiding resource allocation, and developing targeted screening programmes. Existing estimates based on electronic health record (EHR) data suggest a prevalence of 1.4–4.7 cases/million; however, these traditional phenotype-first approaches rely on clinically recognised cases.^5^ The absence of clear diagnostic criteria, variable clinical presentation, overlap with common polygenic metabolic disorders, particularly in partial forms, and lack of systematic genetic testing in routine clinical practice suggest that these phenotype-first studies likely underestimate both the prevalence and phenotypic diversity of monogenic lipodystrophy.^1^, ^2^, ^3^^,^^6^^,^^7^ The significant excess of females in clinically identified cases further supports the ascertainment bias of the phenotype-first studies.^4^^,^^8^^,^^9^

A genotype-first approach, which identifies individuals based on pathogenic variants rather than clinical features, offers a powerful alternative. This method enables the detection of milder or atypical presentations by not relying on the clinical diagnosis of lipodystrophy.^7^^,^^10^, ^11^, ^12^ It takes advantage of clearly defined genetic aetiologies and allows for more accurate estimation of disease prevalence and phenotypic variability, as seen in other monogenic disorders.^7^^,^^11^ A previous genotype-first study of lipodystrophy estimated a prevalence of 1:3,082, but its modest sample size (n = 92,455) and the predominance of a single *LMNA* variant limited generalisability.^6^^,^^7^ The UK Biobank is a large population cohort of ∼500,000 individuals from the UK, with whole genome sequencing and deep phenotypic data.^13^ This provides an excellent opportunity to apply a genotype-first approach in lipodystrophy that overcomes the limitations of phenotype-first studies.

In this study, leveraging the UK Biobank population cohort of ∼500,000 individuals, we aim to estimate the prevalence, phenotypic spectrum, and all-cause mortality associated with monogenic lipodystrophy in the population. We also compare cases identified through the genotype-first approach with those diagnosed via routine clinical care to better understand the phenotypic spectrum of monogenic lipodystrophy.

## Methods

### Study cohorts

#### Population cohort

We used data from the UK Biobank, a large, population-based cohort comprising ∼500,000 individuals from across the UK.^13^ The dataset includes detailed genetic, biochemical, and phenotypic information collected from self-reports, general practice (GP) records, hospital episode statistics, and national death registries.^13^ For this study, we included 490,414 participants with whole genome sequencing (WGS) data. The North West Centre for Research Ethics Committee (11/NW/0382) approved the UK Biobank, and all participants gave written informed consent.

#### Clinically ascertained monogenic lipodystrophy cohort

To compare monogenic lipodystrophy cases identified by the genotype-first approach from the UK Biobank, to those clinically diagnosed in routine clinical care, we included individuals referred to the National Institutes of Health (NIH), USA, after receiving a diagnosis of monogenic lipodystrophy during routine care. The Institutional Review Board of the NIH approved this study (76-DK-0006), and all participants or their guardians provided written informed consent. Where possible, we matched these individuals to UK Biobank participants carrying the same genetic variants.

### Genetic data

We analysed WGS data generated on the Illumina NovaSeq 6000 platform, which achieved an average coverage of 32.5 × (minimum >23.5 × per participant). We aligned sequences to the GRCh38 reference genome and performed variant calling using DRAGEN v3.7.8. These were performed centrally and described in detail recently.^14^

We focused on rare variants in 23 genes associated with monogenic lipodystrophy, as defined by a green or amber rating in the PanelApp.^15^ These genes are *AGPAT2, BLM, BSCL2, CAV1, CAVIN1, CIDEC, EPHX1, FBN1, KCNJ6, LIPE, LMNA, MFN2, MTX2, OTULIN, PCNT, PCYT1A, PIK3R1, PLIN1, POC1A, POLD1, PPARG, WRN*, and *ZMPSTE24*. We annotated all variants using Ensembl’s Variant Effect Predictor (VEP).^16^

For genes with biallelic inheritance, we reviewed rare homozygous missense or protein-truncating variants (gnomAD v2.1.1 MAF <0.001). We were unable to assess compound heterozygous variants due to the lack of parental data. For monoallelic aetiologies, we selected rare heterozygous variants (MAF <0.0001). We also reviewed nonsense, frameshift, and essential splice-site variants classified as high-confidence by LOFTEE.^17^ We classified variants as pathogenic or likely pathogenic using ACMG/ACGS guidelines.^18^^,^^19^ We reviewed all pathogenic variants in IGV to exclude false positives.^20^

Using whole-genome sequencing data, we assessed per-base coverage across all coding exons of the 23 lipodystrophy genes in a random subset of 998 individuals. Twenty two of the 23 genes showed excellent coverage, with more than 94% of coding bases covered at >20× depth in over 90% of individuals and 98% of coding bases were covered at >8× depth in more than 99% of individuals. *PIK3R1* showed the lowest coverage, with 86.59% of coding bases covered at >20 × depth and 93% at >8× depth (Supplementary Table S1). Overall, these data suggest that gene coverage is unlikely to materially affect variant detection in this study.

### Phenotypes generation

We defined study phenotypes using data from hospital episode statistics, primary care records, self-report data, death registries, and cancer registries. We additionally used UK Biobank centrally curated ‘first occurrence’ data to enhance phenotype definition where appropriate. This approach ensured consistent phenotype ascertainment across all conditions. For diabetes, we further supported case definition using baseline HbA1c measurements collected at recruitment. Supplementary Table S2 details the data sources and coding frameworks used for each phenotype.

Disease outcomes were defined using linked hospital episode statistics, primary care records, self-report data, and death registry records, based on validated UK Biobank phenotype definitions (Supplementary Table S2). Liver fibrosis was assessed using the FIB-4 score, calculated from age, aspartate aminotransferase, alanine aminotransferase, and platelet count measured at baseline. Disease onset was defined using the earliest recorded diagnosis from hospital episode statistics, primary care records, self-report, procedure codes, or death registry data. Death registry records contributed a small proportion of cases overall (<5.3%) and were included to maximise case ascertainment, with appropriate censoring applied in survival analyses.

We analysed all-cause mortality data through national death registry linkage, updated to March 2023 centrally by the UK Biobank (details at: https://biobank.ndph.ox.ac.uk/ukb/refer.cgi?id=115559).

Body mass index (BMI) was calculated from height and weight recorded at study enrolment. We defined individuals as taking cholesterol-lowering medication if they self-reported use or held a prescription for statins, fibrates, ezetimibe, or bile acid sequestrants. LDL levels were adjusted for medication use following the method from Klimentidis et al.^21^

The UK Biobank cohort provides DXA-derived total tissue fat percentage in 48,175 cases, and impedance-based body fat percentage in 492,573 cases. Of these, 45,173 participants had both measurements at the same time point. The two measures showed a strong correlation within individuals (R^2^ = 0.86, *P* <2.2 × 10^−16^). In contrast, only DXA-derived total tissue fat percentage was available for clinically ascertained cases. For our primary analysis within UK Biobank, we used impedance-based body fat percentage because of the larger sample size. To enable a valid comparison between UK Biobank cases and clinical cases, which used DXA exclusively, we estimated DXA-equivalent total tissue fat percentages from impedance data using linear regression. This model was developed in 33,879 individuals and validated in 11,294 individuals without lipodystrophy (Supplementary Fig. S1). This correction allowed lipodystrophy cases in UK Biobank with only impedance data to be compared directly with clinically ascertained cases.

The missingness of clinical features assessed in this study from the UK Biobank are reported in Supplementary Table S3.

### Statistical analysis

We calculated 95% confidence intervals for proportions using the Clopper–Pearson method, which provides exact bounds suitable for small or skewed samples. Categorical variables were compared using Fisher’s exact test, and continuous variables using Welch’s t-test.

To assess differences in clinical features by sex, we performed sex-stratified analyses. Continuous traits were summarised as sex-specific means with corresponding confidence intervals and compared using t-tests, while binary outcomes were compared using Fisher’s exact tests, as appropriate. Given the small number of monogenic lipodystrophy cases, sex-stratified descriptive estimates are presented as the primary results. For completeness, we assessed effect modification by sex using unadjusted linear regression that included an interaction term between sex and genotype. Due to the small numbers, these analyses should consider exploratory and interpreted cautiously.

We analysed time-to-event disease outcomes using Cox regression for diabetes, heart failure, coronary artery disease, stroke and hypertension. Outcomes were ascertained at baseline and during follow-up. Because this was a genotype-first study, inclusion was independent of disease status. Individuals with disease diagnosed before recruitment were therefore retained in the analyses. We used the earliest recorded age at diagnosis to define event timing. Follow-up ended at the first occurrence of each outcome, death, emigration, loss to follow-up, or the administrative end of follow-up, whichever occurred first. Participants who did not experience the outcome were censored at the time of death, emigration, loss to follow-up, or administrative end of follow-up. Outcome data were available up to 31 October 2022, and death registry data were available up to 15 March 2023.

Primary analyses adjusted for age at recruitment (continuous), sex (binary), and genetic principal components (continuous) to account for baseline demographic differences and population stratification. We prespecified this parsimonious covariate set because of the genotype-first design and the small number of monogenic lipodystrophy cases, which increased the risk of overfitting in more highly adjusted models.

We assessed the proportional hazards assumption for all Cox models using Schoenfeld residuals (Supplementary Fig. S2). The representative Schoenfeld residual plots are provided in the Supplementary materials. As sensitivity analyses, we repeated analysis using age as the time scale and unadjusted analyses.

To assess the timing of diabetes onset and all-cause mortality using a Kaplan-Meier analysis to estimate an age dependent risk. We used log-rank tests to compare survival curves between groups.

To assess advanced liver fibrosis, we used a previously reported value of Fibrosis-4 (FIB-4) score greater than 2.67, measured at recruitment in UK Biobank.^22^, ^23^, ^24^ As this measure was available at baseline for all participants, we used logistic regression to test the association with pathogenic genotypes, adjusting for age, sex, and genetic principal components.

We performed all analyses using R version 4.4.0.

### Role of funders

The funders were not involved in the study design, data collection, analysis, interpretation, or writing this article.

## Results

### Prevalence of monogenic lipodystrophy

We studied 490,414 UK Biobank participants with whole genome sequencing data to identify individuals with pathogenic genotype in 23 genes linked to total or partial lipodystrophy. We identified 31 individuals with a pathogenic monogenic lipodystrophy genotype, giving a population prevalence of one in 15,820 (0.0063%, 95% CI: 0.0043–0.009%; range: 1 in 23,282–1 in 11,145). The prevalence did not differ significantly between males and females (0.0076% vs. 0.0053%, *P* = 0.37), which contrasts with clinical studies where females are overrepresented.^7^, ^8^, ^9^^,^^25^

Of these, 71% (n = 22) had a pathogenic *PPARG* variant causing familial partial lipodystrophy type 3 (FPLD3); 17 had a protein-truncating variant and 5 had a rare missense variant (Supplementary Table S4). A further 25.8% (n = 8) carried rare pathogenic missense variants in *LMNA*, which causes familial partial lipodystrophy type 2 (FPLD2). One individual had a homozygous pathogenic variant in a *MFN2*, which we cannot report individually due to UK Biobank policy. Notably, none of the individuals with a pathogenic variant had an electronic health record diagnosis of lipodystrophy.

### Clinical features of individuals with a pathogenic monogenic lipodystrophy genotype

To compare carriers of a pathogenic monogenic lipodystrophy variant with non-carriers, we assessed clinical features commonly used to diagnose lipodystrophy in routine practice. Carriers did not differ significantly from non-carriers in age at recruitment (57.6 vs. 57.0 years, *P* = 0.72) or sex distribution (45.2% vs. 54.2% female, *P* = 0.37).

However, carriers had a markedly lower body fat percentage (24.7% vs. 31.4%, *P* = 1.25 × 10^−5^) despite a similar BMI (26.5 vs. 27.4 kg/m^2^, *P* = 0.22). They also had a higher BMI-adjusted waist–hip ratio (0.91 vs. 0.87, *P* = 4.39 × 10^−3^), higher triglyceride levels (2.9 vs. 1.7 mmol/L, *P* = 6.69 × 10^−5^), and lower HDL cholesterol (0.99 vs. 1.45 mmol/L, *P* = 6.26 × 10^−9^). We detected no significant differences in LDL cholesterol, AST, ALT, or GGT (*P* > 0.1) (Table 1). These findings are consistent with the presence of partial lipodystrophy; however, the differences were modest, highlighting the difficulties in identifying these cases in clinical practice. We found no statistically significant differences between carriers of pathogenic variants in *LMNA* and *PPARG* (Supplementary Table S5) or between carriers of *PPARG* protein-truncating and missense variants (Supplementary Table S6), although these analyses were limited by small numbers in each group.

**Table 1:** Clinical features of individuals with monogenic lipodystrophy in the UK Biobank.

Clinical feature	Non-carriers (n = 490,383)	Monogenic lipodystrophy (n = 31)	*P*-value
Age (yrs)	57 (8.1)	57.6 (8.3)	0.72
Females n (%)	265,999 (54.2%)	14 (45.2%)	0.37
BMI (kg/m^2^)	27.4 (4.8)	26.5 (3.9)	0.22
Waist hip ratio BMI adjusted	0.87 (0.08)	0.91 (0.06)	0.0044^a^
Body fat (%)	31.4 (8.5)	24.7 (7)	1.25 × 10^−5^^a^
HDL (mmol/L)	1.45 (0.38)	0.99 (0.27)	6.26 × 10^−9^^a^
LDL^b^ (mmol/L)	3.9 (0.9)	3.9 (1.1)	0.98
Triglycerides (mmol/L)	1.7 (1)	2.9 (1.4)	6.69 × 10^−5^^a^
AST (U/L)	26.2 (10.6)	28.9 (15.6)	0.36
ALT (U/L)	23.5 (14.2)	28.9 (18.2)	0.12
GGT (U/L)	37.4 (42.1)	46.2 (45.9)	0.31

Mean (standard deviation) for Continuous variables and n(%) for categorical variable.

a Bonferroni corrected *P*-value threshold = 0.0045 (0.05/11).

b LDL has been adjusted for cholesterol lowering medication.

In the UK Biobank imaging subset, DXA-derived visceral adipose tissue estimates were available for less than 6 individuals carrying a pathogenic monogenic lipodystrophy variant. Although carriers showed a lower mean visceral adipose tissue mass compared with non-carriers (678 g ± 590 vs. 1226 g ± 939), this difference was not statistically significant, reflecting the very small number of carriers with available imaging data.

### Differences in clinical features by sex

The lack of clinical selection in our cohort and the availability of uniform data for males and females allowed us to examine both within-sex and across-sex differences in the clinical features of lipodystrophy, potentially providing insight into the female enrichment observed in clinical case series.

**Fig. 1:**
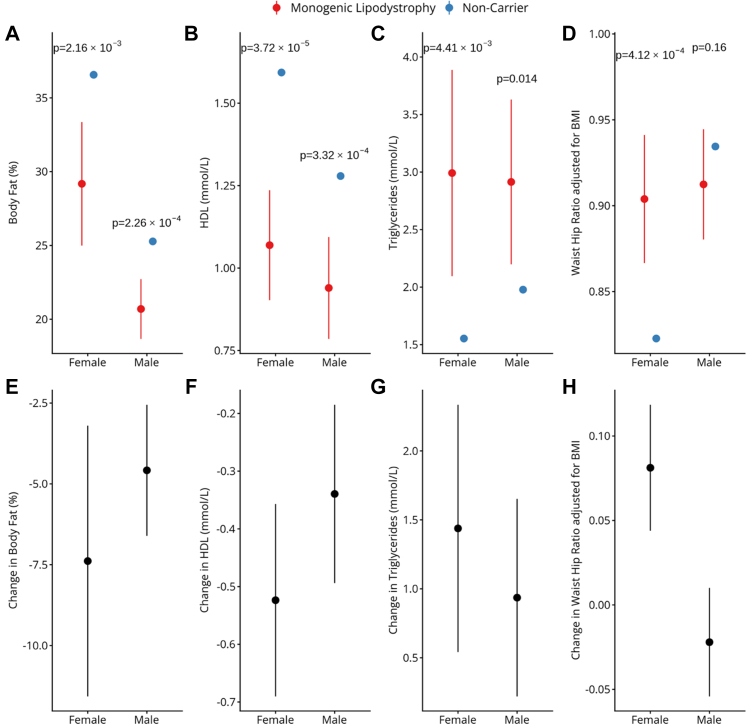
**Key lipodystrophy clinical features by sex and lipodystrophy status and the difference in the clinical features by carrier status.** (A–D) Mean values with 95% CIs are shown for (A) body fat percentage, (B) HDL cholesterol, (C) triglycerides, and (D) waist–hip ratio adjusted for BMI, stratified by sex and carrier status. *P* values are from Wald two-sample t tests comparing carriers and non-carriers within each sex. (E–H) Difference between the means of non-carriers and carriers and 95% CIs stratified by sex for (E) body fat percentage, (F) HDL cholesterol, (G) triglycerides, and (H) waist–hip ratio adjusted for BMI.

Within both sexes, the differences in body fat percentage, HDL cholesterol, and triglycerides between variant carriers and non-carriers were in the same direction as in the combined analysis but the differences were consistently smaller in males (Fig. 1). Compared to non-carriers, body fat percentage in pathogenic variant carriers was reduced by an average of 4.6% in males vs. 7.4% in females; HDL was lower by 0.34 mmol/L in males and 0.52 mmol/L in females; and triglycerides were higher by 0.94 mmol/L in males compared with 1.4 mmol/L in females (Fig. 1B) (Supplementary Table S7). However, due to the small numbers these differences were not statistically significant (*P* interaction sex >0.18).

In contrast, BMI-adjusted waist–hip ratio did not differ from non-carriers in males (0.91 vs. 0.93, *P* = 0.16) but was significantly higher in females (0.90 vs. 0.82, *P* = 4.12 × 10^−4^) (*P* interaction sex = 1.04 × 10^−6^) (Fig. 1D, H). Across-sex comparisons showed that most features were similar between male and female carriers, except for body fat percentage, which was lower in males, and BMI, which was slightly higher in males (20.7 vs. 29.2% body fat; BMI 27.8 vs. 25 kg/m^2^). Together, these findings suggest that clinical features of lipodystrophy differ less from those of non-carriers in males than in females, making affected males harder to identify in clinical practice and therefore more likely to be missed.

### Disease and outcomes in individuals with a monogenic lipodystrophy genotype

**Fig. 2:**
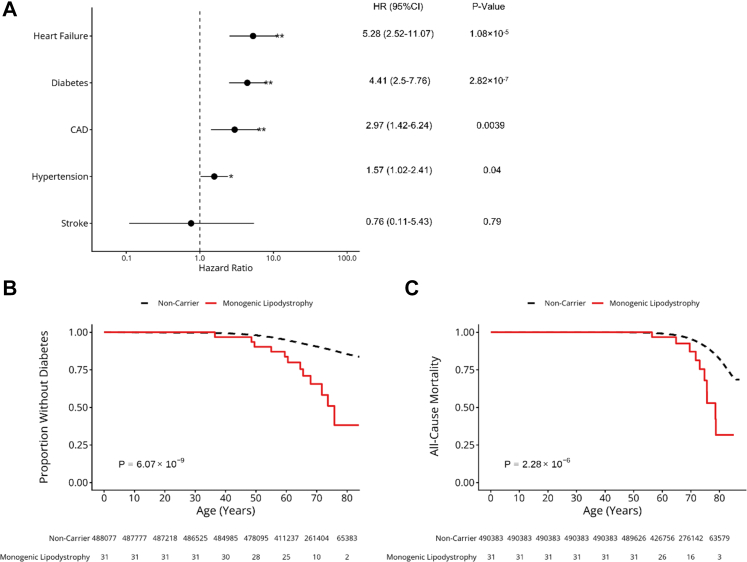
**Association of diabetes, cardiovascular disease and all-cause mortality with monogenic lipodystrophy.** (A) Association of disease outcomes in individuals with a pathogenic monogenic lipodystrophy genotype compared with non-carriers. Hazard ratios were adjusted for age, sex and genetic ancestry principal components. ∗ represents *P* < 0.05 and ∗∗ represents Bonferroni significance (*P* < 0.01) (B) Kaplan–Meier analysis of age-related risk of diabetes in individuals with a pathogenic monogenic lipodystrophy genotype (n = 31) vs. non-carriers (n = 488,077). Log-rank test *P* value is shown. (C) Kaplan–Meier analysis of all-cause mortality in individuals with a pathogenic monogenic lipodystrophy genotype (n = 31) vs. non-carriers (n = 490,383). Log-rank test *P* values are shown.

We next examined whether pathogenic variants increased the risk of metabolic diseases commonly associated with clinically ascertained lipodystrophy. Compared to non-carriers, individuals with a monogenic lipodystrophy genotype had a significantly higher risk of heart failure (HR = 5.28, 95% CI: 2.52–11.07, *P* = 1.08 × 10^−5^), diabetes (HR = 4.41, 95% CI: 2.5–7.76, *P* = 2.82 × 10^−7^), and coronary artery disease (HR = 2.97, 95% CI: 1.42–6.24, *P* = 0.0039) (Fig. 2A, Supplementary Table S8). In contrast, we found no significant enrichment for hypertension (HR = 1.57, 95% CI: 1.02–2.41, *P* = 0.04) or stroke (HR = 0.76, 95% CI: 0.11–5.43, *P* = 0.79). We performed sensitivity analyses using age as a time scale and conducted unadjusted analyses. These analyses produced effect estimates consistent with the primary results supporting the robustness of our findings (Supplementary Tables S9–S11). Additionally, carriers of pathogenic variants in LMNA or PPARG showed directionally consistent results (Supplementary Tables S12 and S13). Finally, we also assessed FIB-4 score at recruitment which was not statistically different from non-carriers (adjusted Odds ratio, 1.01, *P* = 0.81).

Individuals with a pathogenic genotype also developed diabetes at an earlier age than non-carriers (log-rank *P* = 6.23 × 10^−9^). By age 60, 16.3% (95% CI: 2.1–28.3%) of carriers had developed diabetes, compared with 5.1% (95% CI: 5.1–5.2%) of non-carriers (Fig. 2B). Overall, these findings show that despite only modest differences in cross-sectional clinical features, carriers of pathogenic monogenic lipodystrophy variants have a substantially higher risk of metabolic disease.

### Mortality of individuals with a monogenic lipodystrophy genotype

We assessed the impact of lifelong exposure to a pathogenic monogenic lipodystrophy variant on all-cause mortality. Even in this clinically unselected cohort identified through a genotype-first approach, individuals with monogenic lipodystrophy had nearly a four-fold higher risk of death over a mean follow-up of 13.6 years (Adjusted HR 4.02, 95% CI: 2.16–7.48, *P* = 2.35 × 10^−6^). By age 80, 68.3% (95% CI: 25.0–86.6%) of carriers had died, compared with 17.7% (95% CI: 17.6–17.9%) of non-carriers (log-rank *P* = 2.35 × 10^−6^) (Fig. 2C). These findings highlight the substantial impact of lipodystrophy on survival and support early detection and treatment of associated metabolic disorders.

### Comparison of UK biobank and clinically ascertained individuals with monogenic lipodystrophy

**Fig. 3:**
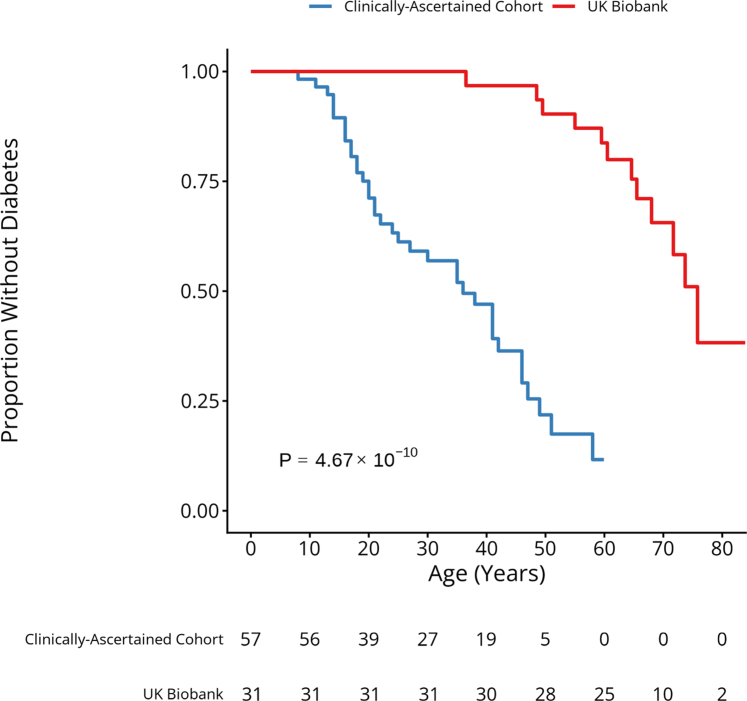
**Age-related risk of diabetes in clinically ascertained vs. UK Biobank monogenic lipodystrophy cases.** Kaplan–Meier analysis of diabetes onset in individuals with monogenic lipodystrophy, stratified by cohort: clinically ascertained cases (n = 57) and UK Biobank cases identified through a genotype-first approach (n = 31). Log-rank test *P* value is shown.

Genotype-first studies in population cohorts often identify individuals with milder phenotypes.^10^^,^^11^ To assess this for lipodystrophy, we compared UK Biobank participants with a pathogenic monogenic lipodystrophy genotype to 58 clinically ascertained cases (4 male, 54 female). Clinically ascertained cases were significantly younger (38 vs. 57.6 years, *P* = 6.6 × 10^−12^) and more often female (93.1% vs. 45.2%, *P* = 9.14 × 10^−7^) than UK Biobank cases (Table 2). They had lower total tissue fat (24.5% vs. 28.7%, *P* = 3.38 × 10^−3^) despite a similar BMI (25.9 vs. 26.5 kg/m^2^, *P* = 0.48). HDL, triglycerides, AST, and ALT levels did not differ significantly (*P* ≥ 0.01). However, LDL cholesterol was significantly lower in the clinically ascertained group (2.5 vs. 3.2 mmol/L, *P* = 7.72 × 10^−4^). Clinically ascertained cases developed diabetes much earlier than UK Biobank cases. By age 50, 78.2% (95% CI: 58.6–88.5%) of clinically ascertained individuals had diabetes, compared with 9.7% (95% CI: 0–19.5%) of UK Biobank cases (log-rank *P* = 4.67 × 10^−10^) (Fig. 3). First, we performed a subgroup analysis restricted to females in both the clinically ascertained cohort and the UK Biobank (n = 54 vs. 14). We then conducted an additional analysis limited to individuals carrying the same variants in the clinical cohort and the UK Biobank (n = 54 vs. 11), to minimise the effect of variant heterogeneity between groups (Supplementary Tables S14 and S15). These findings suggest that although some statistical differences exist between the groups, their clinical features largely overlap. The high rates of diabetes and females in clinical cases highlight the features clinicians rely on to recognise such cases in routine practice.

**Table 2:** Comparison of clinical features of monogenic lipodystrophy cases identified in the UK Biobank (genotype-first, clinically unascertained) and clinically ascertained cases.

Clinical feature	UK Biobank cases (n = 31)	Clinical cases (n = 58)	*P*-value
Age (yrs)	57.6 (8.3)	38 (15)	6.60 × 10^−12^^a^
Females n (%)	14 (45.2%)	54 (93.1%)	9.14 × 10^−7^^a^
Diabetes n (%)	12 (38.7%)	38 (65.5%)	0.024
CAD n (%)	7 (22.6%)	7 (12.1%)	0.24
Cholesterol lowering medications n (%)	12 (38.7%)	31 (53.4%)	0.11
BMI (kg/m^2^)	26.5 (3.9)	25.9 (3.4)	0.48
Total tissue fat (%)	28.7 (6.6)	24.5 (5.1)	3.85 × 10^−3^
HDL (mmol/L)	0.99 (0.27)	0.91 (0.41)	0.27
LDL (mmol/L)	3.2 (0.7)	2.5 (1)	7.72 × 10^−4^^a^
Triglycerides (mmol/L)	2.9 (1.4)	4.7 (5.5)	0.02
AST (U/L)	28.9 (15.6)	26.7 (18.1)	0.56
ALT (U/L)	28.9 (18.2)	39.5 (46.7)	0.14
Pathogenic LMNA variant n (%)	8 (25.8%)	45 (77.6%)	NA
Pathogenic PPARG variant n (%)	22 (71%)	10 (17.2%)	NA

Mean (standard deviation) for Continuous variables and n(%) for categorical variable.

a Bonferroni corrected *P*-value threshold = 0.0038 (0.05/13).

## Discussion

This largest genotype-first study of lipodystrophy using a population cohort shows that monogenic lipodystrophy is rare (1:15,820) but carries a substantial burden of diabetes and cardiovascular disease and a markedly increased risk of all-cause mortality, despite appearing milder in cross-sectional clinical assessment.

Our genotype-first approach produced a minimum prevalence estimate of one in 15,820. This is around tenfold higher than the largest previous UK estimate of 4.7 cases per million and other estimates based on electronic healthcare records (EHR).^5^ This difference is unsurprising, as none of our cases had an EHR diagnosis of lipodystrophy. Our results are consistent with the one other genotype-first study showing higher prevalence estimates than EHR-based approaches.^7^ Interestingly, our genotype-first estimate is about fivefold lower than the genotype first estimate reported by Gonzaga-Jauregui et al. of one in 3082 in DiscovEHR cohort from rural Pennsylvania, USA (n = 92,455).^7^ Their higher estimate was likely influenced by a founder effect of the *LMNA* p.Arg482Gln variant, which accounted for a large proportion of their cases (16/30, 53.3%).^6^^,^^7^^,^^26^ It is also important to note that the UK Biobank consists of generally healthier volunteers, which may reduce the number of individuals with severe disease, as reflected by the absence of generalised lipodystrophy cases. By contrast, the DiscovEHR cohort is healthcare-based, where all participants were consumers of the Geisinger Health System, with many recruited from cardiac catheterisation laboratories and bariatric surgery clinics and therefore are more likely to be enriched for cardiometabolic disorders.^27^^,^^28^ This is supported by the higher mean BMI reported in the DiscovEHR cohort compared with the UK Biobank (31.2 vs. 27.4 kg/m^2^). Taken together, these observations suggest that our estimate represents the minimum prevalence in the general population and is likely applicable to other populations without strong founder effects.

The particular strength of this study is the genotype-first approach in a large cohort. Although we identified only a small number of cases, the uniform data across carriers and non-carriers allowed us to provide novel insights. Previous studies of clinically ascertained cohorts have consistently reported a marked female predominance, suggesting that the disease is more common in women.^7^, ^8^, ^9^^,^^25^ In our cohort, however, we observed no difference in prevalence between males and females. The clinical features of lipodystrophy differ less from those of non-carriers in males than in females, making affected males harder to identify in clinical practice and therefore more likely to be missed. This may explain the female predominance in clinical cohorts, where diagnosis often relies on visual cues of fat loss, which are more pronounced in women (4.6% lower body fat in males vs. 7.4% lower in females compared to non-carriers).^29^ In contrast to previous clinically ascertained series, which reported higher triglycerides, lower HDL, and more diabetes in women, we did not observe such differences.^25^^,^^30^^,^^31^ This likely reflects differences in ascertainment: women in clinical cohorts were usually proband cases, identified because of severe clinical features, while men were more often family members detected through cascade testing, and therefore not selected on phenotype.^25^^,^^30^ Our genotype-first approach reduces this ascertainment bias, removing the apparent sex difference. Nonetheless, within-sex analyses suggested that pathogenic variants had larger effects in females than in males, with little or no impact on BMI-adjusted waist–hip ratio in men. This may reflect the greater volume of adipose tissue in women (36.6 vs. 25.3% body fat), meaning more tissue is qualitatively affected, or a more complex interplay between variants, adipose biology, and reproductive hormones. As the number of individuals with a pathogenic monogenic lipodystrophy variant was low, we may have been underpowered to detect some differences between the sexes. These findings require replication in larger, clinically unselected cohorts to fully understand the sex-specific effects of monogenic lipodystrophy.

Our data strongly support the need for better strategies to detect lipodystrophy. In our study, UK Biobank cases were older, had less diabetes, and carried more body fat than clinically identified cases, suggesting a milder phenotype. Despite this, they still showed a three- to sixfold increase in the risk of cardiometabolic disease outcomes. This may reflect the lifelong impact of even modest cardiometabolic abnormalities caused by monogenic lipodystrophy. These findings strongly suggest that earlier identification and preventative therapy may reduce cardiovascular risk and overall mortality. The lower LDL in clinically ascertained cases may be due to higher statin and other cholesterol lowering medication use. This supports the benefit of earlier diagnosis and treatment. Future research should compare outcomes in monogenic lipodystrophy with those in polygenic forms, which may present later in life and follow a different trajectory.

Our findings support the need for earlier identification of monogenic lipodystrophy, particularly given the availability of effective treatments. An approach analogous to existing clinical risk tools used in monogenic diabetes^32^ could enable targeted genetic testing in individuals at the highest risk. Recently proposed lipodystrophy severity score provides an initial framework^33^; however, larger datasets will be required to develop and validate robust clinical risk models. Integrating data from multiple large population-based biobanks may enable the development of such tools and represents an important direction for future research.

We also found a strong association between monogenic lipodystrophy and heart failure. While this may partly reflect more complete capture of heart failure diagnoses in health records, it may also arise because heart failure can result from both clinically symptomatic and subclinical atherosclerotic disease often observed in lipodystrophy cases.^34^ Heart failure has also been reported in isolated case only series.^35^ Further work is needed to determine whether this reflects a direct effect of *LMNA* and *PPARG* variants on cardiomyocytes, where both genes play important roles in cardiac structure and metabolism, or whether heart failure simply acts as a surrogate marker of broader cardiovascular disease.

This study has limitations. The UK Biobank predominantly includes individuals of European ancestry and healthier volunteers aged 40–70, limiting generalisability and underrepresenting severe early-onset disease. As with all recruitment-based cohort studies, individuals who carried a pathogenic lipodystrophy genotype and died before UK Biobank recruitment could not be included. This survivor bias may lead to underestimation of the true effect size, but it does not introduce immortal time bias, as exposure status was fixed from birth and follow-up time was defined prospectively from recruitment. The use of short-read sequencing and lack of parental data prevented detection of pathogenic compound heterozygous variants, meaning some severe or generalised lipodystrophy cases may have been missed. Our study is limited by the small number of individuals carrying pathogenic monogenic lipodystrophy variants. Although time-to-event analyses suggest earlier diabetes onset overall, confidence intervals overlap at older ages, indicating uncertainty in age-specific prevalence estimates. Larger studies will be required to define age-dependent risk with greater precision. Due to the rarity of monogenic lipodystrophy, even within the largest population-based cohort available, we identified only a small number of individuals carrying pathogenic variants. While this finding is consistent with these genes causing severe human disease, the limited sample size restricted statistical power for detailed subgroup analyses and reduced our ability to detect modest effect associations, including comparisons between *PPARG* and *LMNA* variants and between *PPARG* haploinsufficiency and dominant-negative mechanisms. Additionally, the genotypes of clinically ascertained individuals with lipodystrophy differed from those identified from the UK Biobank with the majority of pathogenic variants in the UK Biobank being PTVs in *PPARG* and the clinically ascertained cases predominantly having pathogenic missense variants in *LMNA* and *PPARG*. This could confound comparisons between the two cohorts. Further studies in independent, clinically unselected cohorts with sequencing data, such as All of Us, will be important to replicate these findings and to assess clinical characteristics, including sex-specific differences. However, the UK Biobank remains the largest population-based cohort of its kind currently available, and analyses in other biobanks are likely to have lower statistical power due to smaller sample sizes. Although our genotype-first design reduces confounding and reverse causation compared with phenotype-first observational analyses, we cannot exclude the possibility of residual confounding. In particular, unmeasured or imperfectly measured factors, including selection and participation biases inherent to UK Biobank. We therefore interpret our findings as evidence of association rather than definitive causal effects.

In conclusion, using a genotype-first approach, we show that monogenic lipodystrophy is rare in the general population and is frequently missed in clinical practice. Despite an apparently milder phenotype, affected individuals remain at high risk of cardiometabolic disease and excess mortality. These findings highlight the importance of early recognition of monogenic lipodystrophy in clinical care and highlight the value of genotype-first approaches for delineating the phenotype spectrum of other underdiagnosed monogenic diseases, enabling earlier diagnosis and timely preventative interventions.

## Contributors

L.N.S researched data, contributed to discussion, and wrote the first draft and subsequent drafts of the manuscript. K.C, J.M.L, A.V.E research data, contributed to discussion, and edited the manuscript. R.J.B provided data from clinically ascertained cases, contributed to discussion, and edited the manuscript. A.T.H, M.N.W contributed to discussion, and edited the manuscript. All authors approved the final version of the manuscript. K.A.P and L.N.S are the guarantors of this work and, as such, had full access to all the data in the study and takes responsibility for the integrity of the data and the accuracy of the data analysis.

## Data sharing statement

UK Biobank dataset is available to researchers from https://biobank.ctsu.ox.ac.uk. The variants used in this study are available in the manuscript. Original data generated and analysed during this study are included in this published article or in the data repositories.

## Declaration of interests

The authors declare that they have no competing interests.
