# Supplementary material for: Genotype-first approach reveals monogenic lipodystrophy is underdiagnosed, with health and mortality risks
Source: eBioMedicine. 2026 Apr 18;127:106255. doi: 10.1016/j.ebiom.2026.106255 (PMC13098338; doi:10.1016/j.ebiom.2026.106255)
Supplement: Supplementary Figs. S1 and S2 and Tables S1–S15 [file mmc1.docx]

**Supplementary Materials**

**Supplemental Table 1 : Coverage of monogenic lipodystrophy genes in 998 randomly selected individuals with whole genome sequencing.**

| Gene | % of bases that have >20 depth in >=90% of individuals | % of bases that have >20 depth in >=95% of individuals | % of bases that have >8 depth in >=99% of individuals |
| --- | --- | --- | --- |
| *AGPAT2* | 99.94 (1546/1547) | 99.16 (1534/1547) | 99.87 (1545/1547) |
| *BLM* | 98.38 (5156/5241) | 86.55 (4536/5241) | 99.96 (5239/5241) |
| *BSCL2* | 99.77 (1706/1710) | 90.82 (1553/1710) | 100 (1710/1710) |
| *CAV1* | 99.51 (2445/2457) | 76.19 (1872/2457) | 99.92 (2455/2457) |
| *CAVIN1* | 99.86 (3566/3571) | 88.32 (3154/3571) | 99.94 (3569/3571) |
| *CIDEC* | 100 (1305/1305) | 88.51 (1155/1305) | 100 (1305/1305) |
| *EPHX1* | 100 (1635/1635) | 82.39 (1347/1635) | 100 (1635/1635) |
| *FBN1* | 99.42 (11542/11609) | 90.53 (10510/11609) | 100 (11609/11609) |
| *KCNJ6* | 94.72 (18658/19698) | 83.22 (16392/19698) | 98.86 (19474/19698) |
| *LIPE* | 99.95 (3769/3771) | 81.91 (3089/3771) | 99.89 (3767/3771) |
| *LMNA* | 99.94 (3177/3179) | 91.38 (2905/3179) | 99.94 (3177/3179) |
| *MFN2* | 99.98 (4407/4408) | 91.33 (4026/4408) | 99.98 (4407/4408) |
| *MTX2* | 100 (1341/1341) | 87.4 (1172/1341) | 100 (1341/1341) |
| *OTULIN* | 99.99 (7969/7970) | 95.42 (7605/7970) | 99.97 (7968/7970) |
| *PCNT* | 99.79 (10506/10528) | 95.35 (10038/10528) | 99.97 (10525/10528) |
| *PCYT1A* | 99.95 (5545/5548) | 92.02 (5105/5548) | 99.95 (5545/5548) |
| *PIK3R1* | 86.59 (6055/6993) | 78.31 (5476/6993) | 93.78 (6558/6993) |
| *PLIN1* | 97.39 (2840/2916) | 83.02 (2421/2916) | 100 (2916/2916) |
| *POC1A* | 100 (1936/1936) | 92.92 (1799/1936) | 100 (1936/1936) |
| *POLD1* | 97.9 (3364/3436) | 97.41 (3347/3436) | 100 (3436/3436) |
| *PPARG* | 100 (1850/1850) | 90.32 (1671/1850) | 100 (1850/1850) |
| *WRN* | 97.19 (7374/7587) | 85.51 (6488/7587) | 99.76 (7569/7587) |
| *ZMPSTE24* | 100 (2975/2975) | 95.03 (2827/2975) | 100 (2975/2975) |

**Supplemental Table 2 : Phenotypes defined in the UK Biobank**

| Clinical Phenotype | Definition |
| --- | --- |
| **Lipodystrophy** | HES record containing a relevant ICD10 code (E88.1, E88.2) or ICD9 code (2726). Individuals were also defined as having lipodystrophy if recorded in their GP records (CTV3: XE11X, X50E8, X76BY, X50EG) (Read2: C326., C3260, C32y6). |
| **Heart Failure** | Self-reported history of Heart Failure or cardiomyopathy (p20002: 1076, 1079); or has a HES record containing a relevant ICD10 code (I11.0,I13.0,I13.2,I50.0,I50.1,I50.9,I25.5,I42.0,I42.5,I42.8,I42.9) or ICD9 code (4254,4280,4281,4289). We additionally included anybody with a relevant first occurrence recorded (I50). This definition is in part based on a definition by Aragam et al 2018 (https://pubmed.ncbi.nlm.nih.gov/30586722/). |
| **Coronary Artery Disease (CAD)** | Self-reported history of CAD (p20002: 1075); or has a HES record containing a relevant ICD10 code (I21.X, I22.X, I23.X, I25.2) or ICD9 code (4109, 4119, 4129) or OPCS4 code (K40.X, K41.X, K45.X, K49.X, K50.2, K75.X). We additionally included anybody with a relevant first occurrence recorded (I21, I22, I23). This definition is in part based on a definition by Aragam et al 2018 (https://pubmed.ncbi.nlm.nih.gov/30586722/). |
| **Hypertension** | Self-reported history of hypertension (p20002: 1065, 1072; p6150_i0/ p6150_i1/ p6150_i2); or has a relevant ICD10 (I10). Individuals were included if they had self-reported being on antihypertensive medication (p20003_i0, p20003_i1, p20003_i2, p20003_i3, p6177_i0, p6177_i1, p6177_i2, p6177_i3, p6153_i0, p6153_i1, p6153_i2, p6153_i3). Individuals were also defined as having hypertension if recorded in their GP records. We additionally included anybody with a relevant first occurrence recorded (I10). Finally, blood pressure was measured, and individuals were classed as being hypertensive if they had a systolic blood pressure > 149 mmHg or a diastolic blood pressure > 90 mmHg. This phenotype is in part based on a definition by Elghazaly et al 2023 (https://pubmed.ncbi.nlm.nih.gov/37309807/). |
| **Stroke** | Self-reported history of stroke (p20002: 1081, 1086, 1491, 1583); or had a relevant ICD10 (I60.X, I61.X, I63.X, I64) or ICD9 (430, 431, 433, 434, 436) code. Individuals were also defined as having a stroke if it was recorded in their GP records. We additionally included anybody with a relevant first occurrence recorded (I60, I61, I63, I64). This phenotype is in part based on a definition by Rannikmäe et al 2022 (https://pubmed.ncbi.nlm.nih.gov/35185750/). |
| **Diabetes** | Self-reported diabetes at any instance (p20002:1222, 1223, 1276, 1468, 1607; p2443, p6148); or self-reported being on any diabetes associated medication (p20003_i0, p20003_i1, p20003_i2, p20003_i3). HbA1c was measured at recruitment and follow up. If they had a HbA1c >= 48mmol/mol then they were classified as having diabetes. We additionally included anybody with a relevant first occurrence recorded (E10, E11, E12, E13, E14). This definition is in part based on definitions by Young et al 2022 (<https://pubmed.ncbi.nlm.nih.gov/35026015/>) and Mirshahi et al 2022 (https://pubmed.ncbi.nlm.nih.gov/36257325/). |
| **Fibrosis-4 (Fib-4) score** | ALT, AST, platelet count and age was used to calculate the fibrosis-4 score. All biochemical measures were collected at recruitment. The following equation by Sterling et al 2006 was used (<https://pubmed.ncbi.nlm.nih.gov/16729309/>):  (Age[years]*AST[U/L])/((platelet count[10^9^/L])*(ALT[U/L])^0.5^) |

**
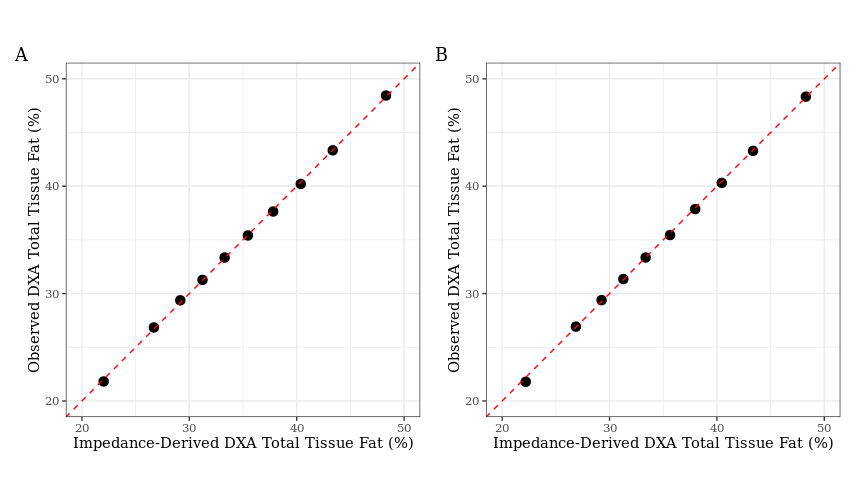
**

**Supplemental Figure 1:** Calibration plots for observed total tissue fat % from DXA against imputed total tissue fat % in the A) Creation cohort (n = 33,880), B) Test cohort (n = 11,294).


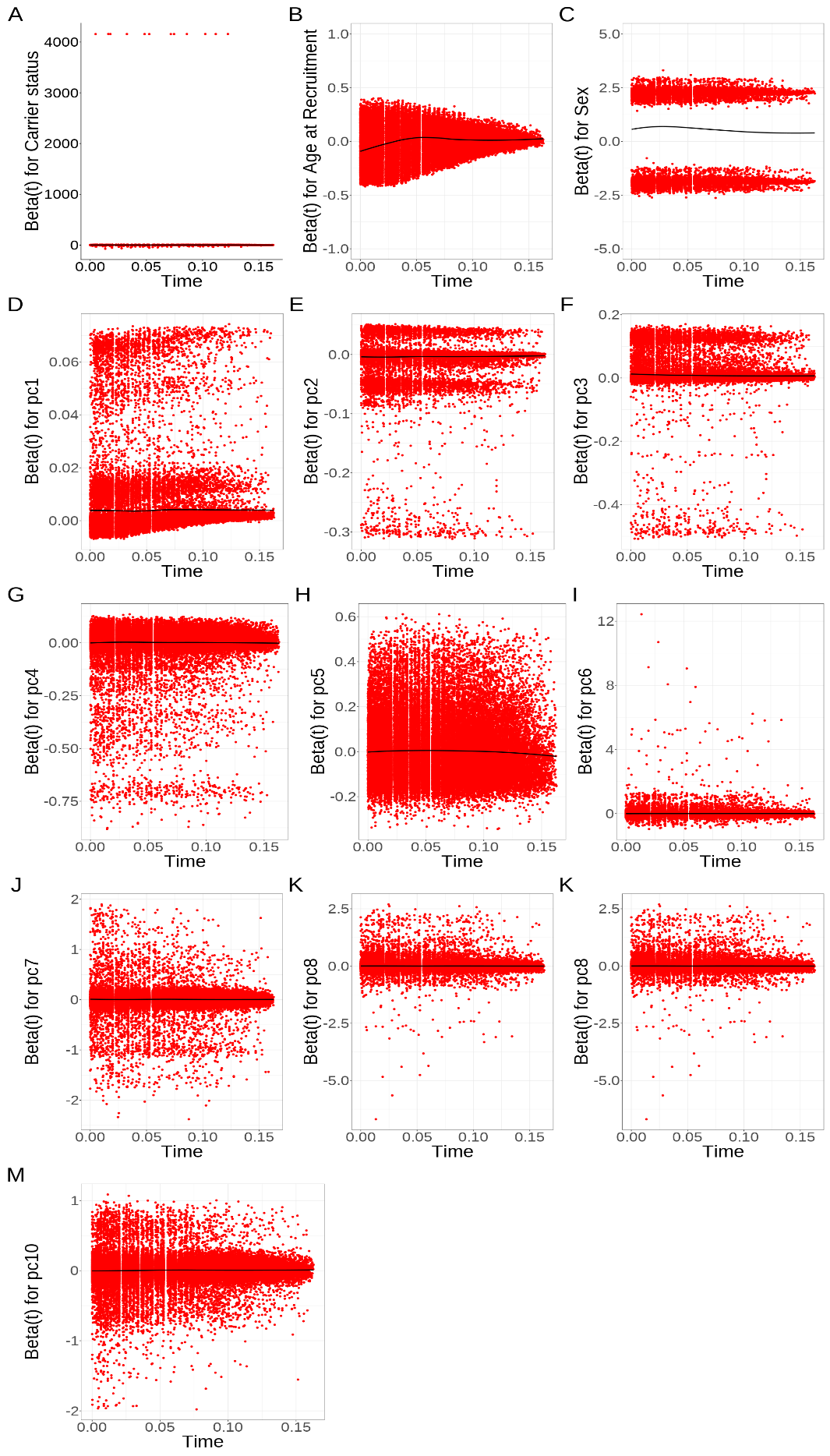
**Supplemental Figure 2: Schoenfeld residual plots for the adjusted Cox proportional hazard model for diabetes.**A) Schoenfeld residual plot for monogenic lipodystrophy carrier status. B) Schoenfeld residual plot for age at recruitment. C) Schoenfeld residual plot for Sex. D-M) Schoenfeld residual plot for genetic ancestry principal component PC1-PC10.

**Supplemental Table 3: Number of individuals with each data on each clinical feature clinical feature by all and sex.**

| **Clinical Feature** | **Monogenic Lipodystrophy (Male/Female)** | **Non-Carriers(Male/Female)** |
| --- | --- | --- |
| Age | **31 (17/14)** | **490383 (224384/265999)** |
| Sex | **31 (17/14)** | **490383 (265999/224384)** |
| BMI | **30 (16/14)** | **488372 (223338/265034)** |
| Waist hip ratio BMI adjusted | **30 (16/14)** | **488382 (223360/265022)** |
| Body Fat | **30 (16/14)** | **481304 (219795/261509)** |
| HDL | **26 (15/11)** | **425252 (196452/228800)** |
| LDL | **29 (16/13)** | **463717 (212327/251390)** |
| Triglycerides | **29 (16/13)** | **464213 (212554/251659)** |
| AST | **29 (16/13)** | **462856 (211930/250926)** |
| ALT | **29 (16/13)** | **464404 (212611/251793)** |
| GGT | **29 (16/13)** | **464346 (212651/251695)** |

**Supplemental Table 4: Variants in *LMNA* and *PPARG* identified in individuals with a pathogenic monogenic lipodystrophy genotype from the UK Biobank.**

| Gene | Protein Change | Nucleotide Change | Classification | Cohort Present In |
| --- | --- | --- | --- | --- |
| *LMNA* | p.Arg482Trp | ENST00000368300.9:c.1444C>T | Pathogenic | Both |
| *LMNA* | p.Arg482Gln | ENST00000368300.9:c.1445G>A | Pathogenic | Both |
| *LMNA* | p.Lys486Asn | ENST00000677389.1:c.1458G>C | Pathogenic | UKBB |
| *PPARG* | p.Glu128Ter | ENST00000287820.10:c.382G>T | Pathogenic | UKBB |
| *PPARG* | p.Cys142Arg | ENST00000287820.10:c.424T>C | Likely Pathogenic | UKBB |
| *PPARG* | p.Tyr192Ter | ENST00000287820.10:c.576C>A | Pathogenic | UKBB |
| *PPARG* | p.Glu217Ter | ENST00000287820.10:c.649G>T | Pathogenic | UKBB |
| *PPARG* | p.Leu246CysfsTer6 | ENST00000287820.10:c.737del | Pathogenic | UKBB |
| *PPARG* | p.Gln299Ter | ENST00000287820.10:c.895C>T | Pathogenic | UKBB |
| *PPARG* | p.Val318Met | ENST00000287820.10:c.952G>A | Likely Pathogenic | UKBB |
| *PPARG* | p.Tyr327Ter | ENST00000287820.10:c.981T>A | Pathogenic | UKBB |
| *PPARG* | p.Tyr327Ter | ENST00000287820.10:c.981T>G | Pathogenic | UKBB |
| *PPARG* | p.Tyr355Ter | ENST00000287820.10:c.1065C>G | Pathogenic | UKBB |
| *PPARG* | p.Glu371Ter | ENST00000287820.10:c.1111G>T | Pathogenic | UKBB |
| *PPARG* | p.Arg385Ter | ENST00000287820.10:c.1153C>T | Pathogenic | UKBB |
| *PPARG* | p.Phe391LeufsTer10 | ENST00000287820.10:c.1170del | Pathogenic | UKBB |
| *PPARG* | p.Arg425Cys | ENST00000287820.10:c.1273C>T | Likely Pathogenic | Both |
| *PPARG* | p.Pro495Leu | ENST00000287820.10:c.1484C>T | Pathogenic | Both |
| *PPARG* | p.Lys94AsnfsTer4 | ENST00000287820.10:c.282del | Pathogenic | NIH |
| *PPARG* | p.Arg194Trp | ENST00000287820.10:c.580C>T | Pathogenic | NIH |
| *PPARG* | p.Gln438Pro | ENST00000287820.10:c.1313A>C | Likely Pathogenic | NIH |
| *PPARG* | p.Ser74Tyrfs*24 | ENST00000287820.10:c.217_220dup | Pathogenic | NIH |

**Supplemental Table 5: Clinical features of LMNA Vs PPARG in the UK Biobank.**

| Clinical Feature | PPARG  (n = 22) | LMNA  (n = 8) | P-Value |
| --- | --- | --- | --- |
| Age (yrs) | 58.2 (8.2) | 55.2 (9.1) | 0.43 |
| Females n (%) | 12 (54.5%) | 2 (25.0%) | 0.23 |
| BMI (kg/m^2^) | 26 (3.4) | 28.4 (4.8) | 0.23 |
| Waist hip ratio BMI adjusted | 0.92 (0.06) | 0.89 (0.06) | 0.21 |
| Body Fat (%) | 26 (7.6) | 22.4 (4.4) | 0.13 |
| HDL (mmol/L) | 1 (0.25) | 0.98 (0.32) | 0.83 |
| LDL** (mmol/L) | 3.7 (0.9) | 4.4 (1.4) | 0.22 |
| Triglycerides (mmol/L) | 3.1 (1.4) | 2.5 (1.4) | 0.32 |
| AST (U/L) | 30.4 (18.1) | 25.4 (7.9) | 0.32 |
| ALT (U/L) | 31.7 (20.5) | 22.5 (10.6) | 0.13 |
| GGT (U/L) | 48.4 (48.7) | 44.2 (43.2) | 0.82 |

Continuous variables are given as a mean and standard deviation and p-value was calculated using a Welch t-test. Binary variables are given as a count and percentage and the p-value was calculated using a Fisher’s exact test. * Bonferroni corrected p-value threshold = 0.0045 (0.05/11). ** LDL has been adjusted for cholesterol lowering medication

**Supplemental Table 6: Clinical features of PPARG Dominant negative Vs Haploinsufficiency in the UK Biobank.**

| Clinical Feature | Haploinsufficiency  (n = 17) | Dominant Negative  (n = 5) | P-Value |
| --- | --- | --- | --- |
| Age (yrs) | 56.5 (7.4) | 63.9 (9.1) | 0.15 |
| Females n (%) | 9 (52.9%) | 3 (60.0%) | 1 |
| BMI (kg/m^2^) | 26.3 (3.5) | 25 (3) | 0.43 |
| Waist hip ratio BMI adjusted | 0.91 (0.06) | 0.97 (0.04) | 0.02 |
| Body Fat (%) | 26.8 (7.7) | 23.2 (7.2) | 0.37 |
| HDL (mmol/L) | 0.95 (0.21) | 1.21 (0.3) | 0.19 |
| LDL** (mmol/L) | 3.6 (0.9) | 3.9 (1.1) | 0.62 |
| Triglycerides (mmol/L) | 3.3 (1.4) | 2.5 (1.6) | 0.35 |
| AST (U/L) | 30.6 (20.7) | 29.8 (7.3) | 0.91 |
| ALT (U/L) | 29.3 (20.6) | 38.9 (20.7) | 0.40 |
| GGT (U/L) | 52.4 (55.2) | 36.6 (19) | 0.35 |

Continuous variables are given as a mean and standard deviation and p-value was calculated using a Welch t-test. Binary variables are given as a count and percentage and the p-value was calculated using a Fisher’s exact test. * Bonferroni corrected p-value threshold = 0.0045 (0.05/11). ** LDL has been adjusted for cholesterol lowering medication

**Supplemental Table 7: Clinical features across and within sex of individuals monogenic lipodystrophy in the UK Biobank.**

| Clinical Feature | Female  Non-Carriers  (n = 265,999) | Female  Monogenic Lipodystrophy  (n = 14) | Male  Non-Carriers  (n = 224,384) | Male  Monogenic Lipodystrophy  (n = 17) | Female  P-Value | Male  P-Value | Monogenic Lipodystrophy: Male vs Female  P-Value |
| --- | --- | --- | --- | --- | --- | --- | --- |
| Age (yrs) | 56.9 (8) | 54.2 (7.9) | 57.2 (8.2) | 60.4 (7.8) | 0.23 | 0.12 | 0.039 |
| Females n (%) | 265,999 (100%) | 14 (100%) | 0 (0%) | 0 (0%) | NA | NA | NA |
| BMI (kg/m^2^) | 27.1 (5.2) | 25 (3.9) | 27.8 (4.2) | 27.8 (3.6) | 0.07 | 1 | 0.049 |
| Waist hip ratio BMI adjusted | 0.82 (0.06) | 0.9 (0.06) | 0.93 (0.05) | 0.91 (0.06) | 4.12x10^-4^* | 0.16 | 0.71 |
| Body Fat (%) | 36.6 (6.9) | 29.2 (7.2) | 25.3 (5.8) | 20.7 (3.8) | 2.16x10^-3^ | 2.26x10^-4^* | 8.92x10^-4^* |
| HDL (mmol/L) | 1.59 (0.38) | 1.07 (0.25) | 1.28 (0.31) | 0.94 (0.28) | 3.72x10^-5^* | 3.32x10^-4^* | 0.22 |
| LDL** (mmol/L) | 3.9 (0.9) | 3.6 (0.8) | 3.9 (0.9) | 4.1 (1.3) | 0.26 | 0.52 | 0.23 |
| Triglycerides (mmol/L) | 1.6 (0.9) | 3 (1.5) | 2 (1.2) | 2.9 (1.3) | 4.41x10^-3^ | 0.01 | 0.89 |
| AST (U/L) | 24.5 (9.6) | 26 (6.8) | 28.2 (11.4) | 31.3 (20.1) | 0.46 | 0.55 | 0.33 |
| ALT (U/L) | 20.2 (12.2) | 27.3 (13.5) | 27.5 (15.3) | 30.3 (21.7) | 0.08 | 0.61 | 0.65 |
| GGT (U/L) | 30.3 (33.7) | 38.9 (45.4) | 45.8 (48.9) | 52.2 (46.9) | 0.51 | 0.59 | 0.45 |

Continuous variables are given as a mean and standard deviation and p-value was calculated using a Welch t-test. Binary variables are given as a count and percentage, and the p-value was calculated using a Fisher’s exact test. * Bonferroni corrected p-value threshold = 0.0017(0.05/30). ** LDL has been adjusted for cholesterol lowering medication.

**Supplemental Table 8: Adjusted hazard ratio and counts for individuals included in model**

| End Organ Damage | Adjusted Hazard Ratio (95%CI) | P-Value | N carriers included | N non-carriers included |
| --- | --- | --- | --- | --- |
| Heart Failure | 5.28 (2.52-11.07) | 1.08×10^-5^* | 31 | 485155 |
| Diabetes | 4.41 (2.5-7.76) | 2.82×10^-7^* | 31 | 482930 |
| CAD | 2.97 (1.42-6.24) | 0.0039* | 31 | 484893 |
| Hypertension | 1.57 (1.02-2.41) | 0.04 | 30 | 473648 |
| Stroke | 0.76 (0.11-5.43) | 0.79 | 31 | 485083 |

Hazard ratios are from an adjusted cox proportional hazard model adjusted for: sex, age and genetic ancestry principal components. Each outcome was measured using a separate cox regression. * Values are below Bonferroni significance threshold of 0.01 (0.05/5).

**Supplemental Table 9: Unadjusted hazard ratio and counts for individuals included in model**

| End Organ Damage | Unadjusted Hazard Ratio (95%CI) | P-Value | N carriers included | N non-carriers included |
| --- | --- | --- | --- | --- |
| Heart Failure | 5.59 (2.66-11.72) | 5.34×10^-6^* | 31 | 490330 |
| Diabetes | 4.6 (2.61-8.1) | 1.24×10^-7^* | 31 | 488077 |
| CAD | 3.41 (1.63-7.16) | 0.0012* | 31 | 490068 |
| Hypertension | 1.66 (1.08-2.55) | 0.02 | 30 | 478664 |
| Stroke | 0.79 (0.11-5.63) | 0.82 | 31 | 490255 |

Hazard ratios are from an unadjusted cox proportional hazard model. Each outcome was measured using a separate cox regression. * Values are below Bonferroni significance threshold of 0.01 (0.05/5).

**Supplemental Table 10: Adjusted hazard ratio and counts for individuals included in model using a time scale approach**

| End Organ Damage | Adjusted Hazard Ratio (95%CI) | P-Value | N carriers included | N non-carriers included |
| --- | --- | --- | --- | --- |
| Heart Failure | 5.45 (2.46-12.21) | 3.06×10^-5^* | 30 | 482081 |
| Diabetes | 4.46 (1.86-10.73) | 8.26×10^-4^* | 24 | 457724 |
| CAD | 3.33 (1.25-8.86) | 0.016 | 28 | 470200 |
| Hypertension | 1.21 (0.46-3.24) | 0.7 | 13 | 300579 |

Hazard ratios are from an adjusted cox proportional hazard model adjusted for: sex, age and genetic ancestry principal components. Each outcome was measured using a separate cox regression. Stroke was excluded from the analysis due to no carriers having a stroke after recruitment. * Values are below Bonferroni significance threshold of 0.01 (0.05/5).

**Supplemental Table 11: Unadjusted hazard ratio and counts for individuals included in model using a time scale approach**

| End Organ Damage | Unadjusted Hazard Ratio (95%CI) | P-Value | N carriers included | N non-carriers included |
| --- | --- | --- | --- | --- |
| Heart Failure | 5.84 (2.62-13) | 1.55×10^-5^* | 30 | 487229 |
| Diabetes | 4.51 (1.88-10.84) | 7.53×10^-4^* | 24 | 462625 |
| CAD | 3.55 (1.33-9.45) | 0.01 | 28 | 475238 |
| Hypertension | 1.28 (0.48-3.42) | 0.62 | 13 | 303824 |

Hazard ratios are from an unadjusted cox proportional hazard model. Each outcome was measured using a separate cox regression. Stroke was excluded from the analysis due to no carriers having a stroke after recruitment. *Values are below Bonferroni significance threshold of 0.01 (0.05/5).

**Supplemental Table 12: Unadjusted hazard ratio and counts for individuals included in model for individual with a pathogenic variant in LMNA against non-carriers**

| End Organ Damage | Adjusted Hazard Ratio (95%CI) | P-Value | N carriers included | N non-carriers included |
| --- | --- | --- | --- | --- |
| Heart Failure | 18.73 (6.04-58.08) | 3.89×10^-7^* | 8 | 490330 |
| Diabetes | 5.96 (1.92-18.49) | 0.002* | 8 | 488077 |
| CAD | 8.08 (2.61-25.06) | 2.95×10^-4^* | 8 | 490068 |
| Hypertension | 2.52 (1.05-6.06) | 0.04 | 7 | 478664 |

Hazard ratios are from an unadjusted cox proportional hazard models. Each outcome was measured using a separate cox regression. * Values are below Bonferroni significance threshold of 0.01 (0.05/5). Stroke was excluded from this analysis due to no carriers having had a stroke.

**Supplemental Table 13: Unadjusted hazard ratio and counts for individuals included in model for individual with a pathogenic variant in PPARG against non-carriers**

| End Organ Damage | Adjusted Hazard Ratio (95%CI) | P-Value | N carriers included | N non-carriers included |
| --- | --- | --- | --- | --- |
| Heart Failure | 2.96 (0.95-9.17) | 0.06 | 22 | 490330 |
| Diabetes | 4.05 (2.03-8.11) | 7.53×10^-5^* | 22 | 488077 |
| CAD | 1.85 (0.6-5.74) | 0.29 | 22 | 490068 |
| Hypertension | 1.47 (0.89-2.44) | 0.14 | 22 | 478664 |
| Stroke | 1.06 (0.15-7.5) | 0.96 | 22 | 490255 |

Hazard ratios are from an unadjusted cox proportional hazard models. Each outcome was measured using a separate cox regression. * Values are below Bonferroni significance threshold of 0.01 (0.05/5).

**Supplemental Table 14: Comparison of clinical features against clinically ascertained individuals in females**

| Clinical Feature | UK Biobank Cases  (n = 14) | Clinical Cases  (n = 54) | P-Value |
| --- | --- | --- | --- |
| Age (yrs) | 54.2 (7.9) | 38.3 (14.6) | 2.71×10^-6^* |
| Females n (%) | 14 (100%) | 54 (100%) | NA |
| Diabetes n (%) | 4 (28.6%) | 37 (68.5%) | 0.005 |
| CAD n (%) | 0 (0%) | 6 (11.1%) | 0.33 |
| Cholesterol lowering medications n (%) | 3 (21.4%) | 30 (55.6%) | 0.014 |
| BMI (kg/m^2^) | 25 (3.9) | 25.6 (3.3) | 0.59 |
| Total Tissue Fat (%) | 33 (6.8) | 24.4 (5) | 4.03×10^-4^* |
| HDL (mmol/L) | 1.07 (0.25) | 0.92 (0.42) | 0.14 |
| LDL (mmol/L) | 3.3 (0.9) | 2.5 (1) | 0.01 |
| Triglycerides (mmol/L) | 3 (1.5) | 4.9 (5.6) | 0.03 |
| AST (U/L) | 26 (6.8) | 26.8 (18.7) | 0.8 |
| ALT (U/L) | 27.3 (13.5) | 39.4 (48.3) | 0.11 |
| Pathogenic LMNA variant n (%) | 2 (14.3%) | 42 (77.8%) | NA |
| Pathogenic PPARG variant n (%) | 12 (85.7%) | 9 (16.7%) | NA |

Continuous variables are given as a mean and standard deviation and p-value was calculated using a Welch t-test. Binary variables are given as a count and percentage and the p-value was calculated using a Fisher’s exact test. * Bonferroni corrected p-value threshold = 0.0038 (0.05/13).

**Supplemental Table 15: Comparison of clinical features against clinically ascertained individuals in individuals with matched variants between the cohorts.**

| Clinical Feature | UK Biobank Cases  (n = 11) | Clinical Cases  (n = 54) | P-Value |
| --- | --- | --- | --- |
| Age (yrs) | 57 (9.7) | 37.7 (15.2) | 2.42×10^-5^* |
| Females n (%) | 3 (27.3%) | 50 (92.6%) | 1.33×10^-5^* |
| Diabetes n (%) | 5 (45.5%) | 34 (63.0%) | 0.32 |
| CAD n (%) | 5 (45.5%) | 5 (9.3%) | 0.011 |
| Cholesterol lowering medications n (%) | 6 (54.5%) | 28 (51.9%) | 1 |
| BMI (kg/m^2^) | 27.2 (4.9) | 25.9 (3.5) | 0.4 |
| Total Tissue Fat (%) | 25.3 (4.5) | 24.1 (4.9) | 0.43 |
| HDL (mmol/L) | 0.99 (0.29) | 0.93 (0.42) | 0.6 |
| LDL (mmol/L) | 3.2 (0.7) | 2.6 (1) | 0.03 |
| Triglycerides (mmol/L) | 2.7 (1.4) | 4.7 (5.6) | 0.03 |
| AST (U/L) | 27.8 (7.7) | 26.5 (18.5) | 0.7 |
| ALT (U/L) | 27.1 (16.4) | 39.6 (48.3) | 0.14 |
| Pathogenic LMNA variant n (%) | 7 (63.6%) | 45 (83.3%) | NA |
| Pathogenic PPARG variant n (%) | 3 (9.1%) | 6 (11.1%) | NA |

Continuous variables are given as a mean and standard deviation and p-value was calculated using a Welch t-test. Binary variables are given as a count and percentage, and the p-value was calculated using a Fisher’s exact test. * Bonferroni corrected p-value threshold = 0.0038 (0.05/13).
